# Physiological investigation of arbuscular mycorrhizal fungi-mediated salt tolerance in persimmon (*Diospyros kaki*) seedlings

**DOI:** 10.3389/fpls.2026.1924456

**Published:** 2026-09-23

**Authors:** Berken Cimen, Meral Incesu, Ibrahim Ortas, Mujgan Guney

**Affiliations:** 1 Department of Horticulture, Faculty of Agriculture, Cukurova University, Adana, Türkiye; 2 Department of Soil Science, Faculty of Agriculture, Cukurova University, Adana, Türkiye; 3 Department of Horticulture, Faculty of Agriculture, Yozgat Bozok University, Yozgat, Türkiye

**Keywords:** arbuscular mycorrhizal fungi, *Diospyros kaki*, plant growth, plant nutrient, salinity stress

## Abstract

**Introduction:**

Climate change has increased the frequency of drought periods in the Mediterranean basin, including the Çukurova region, and is projected to exacerbate both drought stress and soil salinization in future agricultural systems. Arbuscular mycorrhizal fungi (AMF) are known to enhance plant growth and alleviate abiotic stress, yet their potential to improve salt tolerance in *Diospyros kaki*, a species considered sensitive to salinity, remains underexplored.

**Methods:**

This study, evaluated the effects of two AMF species, *Entrophospora etunicatum* and *Glomus clarum*, on growth parameters, chlorophyll content, photosystem II efficiency (*Fv’*/*Fm’*), and leaf and root concentrations of K^+^, P, Ca^2+^, Na^+^, and Cl^-^ in *D. kaki* seedlings exposed to five NaCl concentrations (0, 50, 75, 100, and 150 mM).

**Results and Discussion:**

Both AMF species, particularly *E. etunicatum*, significantly improved plant growth, chlorophyll content, and *Fv’*/*Fm’* ratio under saline conditions. Although salt stress alone markedly reduced vegetative growth, the salt × mycorrhiza interaction was not statistically significant for growth parameters, suggesting that AMF-mediated growth promotion occurred independently of salinity level. However, for most mineral nutrients, mycorrhizal inoculation significantly increased tissue concentrations, and the salt × mycorrhiza interaction was statistically significant, indicating that AMF enhanced tissue nutrient concentration specifically under salt stress. These findings support Sustainable Development Goals 2 (Zero Hunger) and 13 (Climate Action) by offering a biological strategy to sustain persimmon production in salinizing environments.

## 1 Introduction

*Diospyros kaki* (persimmon) originated in China, with its cultivation in Western countries commencing only in the second half of the 19th century. In recent decades, persimmon cultivation has garnered significant interest across the Mediterranean basin, including Türkiye (Incesu et al., 2014; Gil-Muñoz et al., 2020). While this region possesses fertile soils conducive to the cultivation of various subtropical species, such as olive, citrus, fig, banana, and avocado, it is increasingly confronting the challenges of climate change. Diminishing rainfall and escalating soil salinity, particularly in coastal areas, have emerged as critical constraints on agricultural productivity. The use of rootstock has become a pivotal strategy to mitigate such edaphic stresses and expand the use of agricultural land.

Within the Diospyros genus, three species are predominantly employed as rootstocks for persimmon: *D. kaki*, *D. lotus*, and *D. virginiana* (Badenes et al., 2015). Although *D. kaki* is the most widely used rootstock globally due to its superior compatibility with commercial cultivars (Bellini and Giordani, 2002), its application in the Mediterranean basin is limited by its sensitivity to calcareous soils, where it develops a weak root system leading to poor growth. In contrast, *D. kaki* remains the predominant rootstock in Turkish persimmon production (Incesu et al., 2014). Salinity stress is an escalating concern in Adana, a key persimmon-producing region, as it adversely affects plant growth and development, representing one of the most significant abiotic stressors limiting yield and quality. This issue is global, threatening 20% of agricultural lands, with projections indicating that this figure could reach 50% within the next two decades (Çimrin et al., 2020). Consequently, as persimmon cultivation expands across the Mediterranean basin, the development of effective salinity mitigation strategies is imperative. The primary approach involves deploying salt-tolerant cultivars and rootstocks, with existing research identifying *D. virginiana* as the most salt-tolerant species, *D. lotus* as the most sensitive, and *D. kaki* as intermediate (Gil-Muñoz et al., 2020).

AMF constitute a vital component of soil ecosystems, forming symbiotic associations with over 70% of terrestrial plants (Janoušková et al., 2013; Zeng et al, 2025). This symbiosis enhances host plant uptake of water and minerals in return for photosynthetic carbon, and a substantial body of evidence indicates that AMF inoculation can alleviate salt-induced toxicity by regulating ion homeostasis, improving nutrient acquisition, and restoring water balance (Carretero et al., 2008; Porcel et al., 2012; Hameed et al., 2014; Dar et al., 2023). The positive effects of AMF on growth and stress tolerance have been documented across a wide range of horticultural crops—including eggplant, zucchini, tomato, pepper, citrus, and strawberry—under saline conditions (e.g., Colla et al., 2008; Kaya et al., 2009; Khalil et al., 2011; Mohammad and Mittra, 2013; Beltrano et al., 2013; Elhindi et al., 2017; Yu et al., 2022).

Despite this extensive body of research, the specific interaction between AMF and persimmon rootstocks under salinity stress remains conspicuously underexplored. To date, only a handful of studies have examined AMF colonization in *Diospyros* species (Incesu et al., 2015; Machineski et al., 2018), and none have systematically investigated whether mycorrhizal inoculation can enhance the salt tolerance of *D. kaki* rootstocks—the very rootstock most widely used in Turkish persimmon orchards yet most vulnerable to the region’s rising soil salinity. This knowledge gap is particularly critical given the Mediterranean basin’s increasing salinization and the absence of practical, sustainable solutions tailored to persimmon production.

In response to the increasing threat of salinity to persimmon production in the Mediterranean region, this study aims to evaluate the efficacy of two AMF species, *E. etunicatum* and *G. clarium*, on the performance of *D. kaki* rootstocks under varying levels of NaCl-induced salinity (0, 50, 75, 100, and 150 mM). Plant growth parameters, chlorophyll content, photosystem II (PSII) activity, and nutrient distribution were assessed to identify optimal mycorrhizal treatments for sustaining productivity in salt-affected orchards.

## 2 Materials and methods

### 2.1 Plant material and raising seedlings

Seeds of *D. kaki* L. were obtained from Cukurova University, Faculty of Agriculture, Department of Horticulture, Persimmon Germplasm Orchard. The experiment was carried out in greenhouse conditions at the Cukurova University Research Farm (37° 1’51.26”N, 35°22’4.43”E) in Adana, Turkey. The mycorrhizal and non-mycorrhizal plants were cultivated for eight weeks in a greenhouse maintained at 25–28 °C with 70–80% relative humidity. They were exposed to a 16-hour light and 8-hour dark photoperiod at a light intensity of 50 µmol m^-^² s^-^¹, and distilled water was supplied regularly to keep soil moisture at 80% of field capacity. Pots were surface-sterilized with ethanol before being filled with growth media. *D. kaki* L. was used as the control treatment. *D. kaki* seeds were surface-sterilized with sodium hypochlorite solution (1% active chlorine) for 10 min, rinsed three times, and then soaked in distilled water several times.

### 2.2 AMF inoculation and salinity treatments

Eight weeks after sowing *D. kaki* seeds, inoculated and non-inoculated uniform seedlings at the three- to four-true-leaf stage with the AMF species were transplanted into three L plastic pots as described in Ortas et al. (2011). Each seedling was transplanted into a single pot. The pots were periodically and manually watered every 2–3 days to keep the soil moisture at field capacity. The plants were grown in a greenhouse at 30–35 °C and 70–85% relative humidity, with a 16 h day and 8 h dark photoperiod. After inoculating the plants with AMF, they were treated with five different salinity levels (0 mM, 50 mM, 75 mM, 100 mM, and 150 mM NaCl) when they were six months old. Stress conditions were maintained for 70 days as described in Incesu et al (Incesu et al., 2014). NaCl was dissolved directly in the irrigation water and applied gradually, with the target concentration for each salinity level reached incrementally over successive irrigations every one a week, to avoid osmotic shock. Pots were free-draining with no leaching. During this period, plants were irrigated with 500 ml water once a week. At the end of the experiment, the severity of leaf symptoms was ranked as follows: 1 - normal healthy green plants without any injury; 2 - slightly wilted, damages on leaf tip; 3 - moderate to severe damages on leaves (70% of defoliated leaves); 4 - most leaves with drying damage (90% of defoliated leaves). 1000 spores from each mycorrhizal inoculum per plant. *G. etunicatum* (Becker & Gerdemann) and *G. clarium* (Nicolson & Schenck) mycorrhizal species, together with non-inoculated plants, were used, and the medium was inoculated 3 cm below the seedling roots. The same amount of non-mycorrhizal sterilized inoculum was applied to pots that were not inoculated. The mycorrhizal species used in the experiment were tested for efficacy before use. Inocula were obtained from the Bank of European Glomales database, INRAE-France, and propagated from sorghum plant roots in trap culture medium.

The experiment was performed in an andesitic tuff, soil, and compost (6:3:1 v/v) mixture (Satir et al., 2016). Soil material was collected from the surface horizons of the clay loam Menzilat soil series (0–20cm) in the Cukurova Basin (South Turkey). Soil pH was 7.45, low organic matter content (1.41%), high CaCO_3_ content (28%), and 0,5 M NaHCO_3_ (pH=8.5) extractable 4.55 kg da^-1^ phosphorus. Prior to the experiment, half of the soil was sterilized in an autoclave at 120 °C for 2 hours. Following sterilization, the soil was allowed to rest for 18 days before being used as the growth medium. 3-L container pots were used under greenhouse conditions.

### 2.3 Leaf chlorophyll concentration and chlorophyll fluorescence measurements

The concentrations of chlorophyll per area were estimated in the same attached leaves as those for which gas exchange measurements were taken using a SPAD portable apparatus (Minolta Co., Osaka, Japan). SPAD readings were used to estimate leaf Chl concentration because there is a strong relationship between SPAD readings and chlorophyll levels (Jifon et al., 2005). Chlorophyll fluorescence parameter (*Fv’*/*Fm’*) was measured with a portable fluorimeter (Photon System Instruments Ltd). Readings were recorded at the two youngest fully expanded leaves of each replicate (Incesu et al., 2014).

### 2.4 Growth parameters and root colonization

After SPAD, chlorophyll fluorescence measurements, the plants were harvested, and leaf number and stem length per plant were measured. Furthermore, the plants were separated into shoots (all leaves and stems) and roots for dry matter assimilation and dried at 72 °C until the weights were stabilized using a thermo-ventilated oven. At harvest dry weights of shoot and root were recorded (Incesu et al., 2014). Collected roots were separated from the growth medium by washing with running tap water and distilled water. Roots were dried on tissue paper. Before drying, small sub-samples were taken from roots and preserved in a mixture of ethanol, glacial acetic acid, and formalin for the determination of root length and mycorrhizal infection. Root clearing, staining, and the extent of cortical mycorrhizal infection were evaluated following the protocol of Koske and Gemma (1989). Root colonization was assessed on all 150 plants, examining 100 root intersections per root system using the gridline intersect method (Giovannetti and Mosse, 1980).

### 2.5 Leaf and root mineral analysis

At the end of the experiment, plants were removed from the pots, separated into roots and shoots, and washed with deionized water. Roots and shoots were oven-dried for two days at 72 °C. The dry material was ground. Chloride concentration was determined by using a scientific chloride analyzer. Dried leaf and root samples were ground to a fine powder using a ball mill. A 0.125 g subsample of the powdered tissue was extracted overnight at room temperature with 10 mL of a solution containing 0.1 N nitric acid (HNO_3_) in 10% (v/v) glacial acetic acid. Following incubation, the mixture was centrifuged and the supernatant was collected. An aliquot of the clear extract (0.5 mL) was subsequently analyzed for chloride concentration using a Sherwood 926 Chloride Analyzer. K, Ca. Zn and Na were determined by atomic absorption spectrophotometry. A 200 mg aliquot of the dried and ground leaf and root samples was ashed in a furnace at 550 °C for 8 hours. Following the ashing process, the resultant ash was dissolved in 1/3 (v/v) HCl and filtered through a blue-band filter paper. The concentrations of K, Ca, Zn, and NA were then determined using an Atomic Absorption Spectrometer (Murphy and Riley, 1962). P concentration was analyzed with a spectrophotometer. A 200 mg aliquot of the dried and ground leaf samples was ashed in a furnace at 550 °C for 8 hours. Following the ashing process, the resultant ash was dissolved in 1/3 (v/v) HCl and filtered through a blue-band filter paper. From the resulting solution, a mixture was prepared containing 0.5 µL of the sample solution, 0.5 µL of Barton’s reagent, and 4 mL of distilled water, which was then vortexed. The vortexed samples were placed into plates and read spectrophotometrically at a wavelength of 430 nm.

### 2.6 Statistical analysis

The experiment was arranged as in a ‘Randomized Complete Block Design’ were the model used: Trait ~ Block + AMF + Salinity treatment + AMF × Salinity treatment (Type II sums of squares), treating Block as the RCBD blocking factor. The experiment used a total of 150 plants, arranged as a randomized complete block design with 10 blocks. Each block contained one plant per treatment combination (3 inoculation treatments × 5 salinity levels = 15 combinations per block; 15 × 10 blocks = 150 plants). Each pot (one plant) represented a single, independent experimental unit, and pots were randomized within each block within the greenhouse. Data were subjected to a two-way ANOVA. Means were computed for each parameter. When the F-test indicated significant treatment effects at (*P* < 0.05), mean comparisons among rootstocks were performed using Fisher’s least significant difference (LSD) test. In addition, the correlation coefficients between all measured parameters were calculated. All statistical analyses were conducted using R (version 4.6.1), while data visualization was carried out using RStudio (version 2026.07.0, packages: agricolae, ggplot2, dplyr, xlsx, predict3d, tidyr).

## 3 Results

### 3.1 Root colonization and growth parameters

Plant root colonization varied by mycorrhizal inoculation from 29.5% to 71.0% in terms of salinity treatments. Increasing salinity levels had reduced the AMF root colonization (%). Since the growth medium was sterilized, the control treatment had no infection (**Table 1**). A two-way ANOVA indicated a significant AMF inoculation treatment main effect (*p* < 0.05) for nearly every investigated variable. In addition, salinity treatment’s main effect was significant for most investigated variables too. AMF × salinity interaction was significant for leaf number, symptom score, leaf Cl concentration, root Cl concentration, leaf calcium concentration, leaf K concentration, leaf Na concentration, leaf Zn concentration, root Ca concentration, root K concentration, root Na concentration, root P concentration and root Zn concentration — meaning the salt-dose response depends on which AMF treatment was applied for those. In contrast, block effect was never significant. Leaf Cl concentration had the highest *R*^2^, interpreting that 96% of the variation in leaf chlorine is explained by AMF, salinity treatment, their interaction, and block (**Table 2**). Main effects of mycorrhiza and salinity treatments were significant (*p ≤* 0.01) on fresh root weight (g). In the mycorrhiza treatment, the highest root weight was observed in plants inoculated with *G. clarium* (12.66 g) and *E. etunicatum* (14.25 g), whereas the lowest fresh weight was in the control (4.47 g). In salt applications, the highest root fresh weight was observed with 0 mM NaCl (12.82 g), and root fresh weight decreased with increasing salt doses (**Table 3**). The effect of different mycorrhiza treatments on fresh stem weight was significant, whereas salt dose and the salt × mycorrhiza interaction were not. The highest fresh stem weight was obtained in *G. clarium* (6.25 g) and *E. etunicatum* (7.14 g), and the lowest in control plants (2.26 g) across mycorrhiza treatments. The main effect of inoculation treatments and salt application on dry root weight was statistically significant. Regarding inoculation treatment, the highest dry root weight was observed in *G. clarium* (2.60 g) and *E. etunicatum* (2.89 g), whereas it was the lowest in the control (1.19 g). Inoculation treatments were significant for root dry weight. The highest dry root weight was 2.82 g in the 0 mM NaCl treatment, and the lowest dry root weight was determined in the 150 mM NaCl treatment (1.70 g). The main effects of three inoculation treatments and salt application on stem dry weight were statistically significant, whereas their interaction was not (**Table 3**). Regarding mycorrhiza treatment, the highest stem dry weight was observed in *G. clarium* (1.54 g) and *E. etunicatum* (1.61 g), and the lowest in the control (0.50 g). The highest dry weight of the stem (1.49 g) was determined in the 0 mM NaCl treatment, and the lowest dry root weight was obtained in the 150 mM NaCl-treated plants (0.92 g). A two-way ANOVA indicated significant main effects of mycorrhiza and salinity treatments and their interaction effect were significant (*p ≤* 0.01) on the number of leaves. For the mycorrhiza treatment, the highest number of leaves was obtained in *G. clarium* (11.94) and *E. etunicatum* (11.31). The number of leaves was the lowest in control plants (6.97). It was determined that mycorrhiza applications increased leaf number. In salt applications, the highest number of leaves was observed with 0 mM NaCl (12.10), 50 mM NaCl (11.10), and 75 mM NaCl (10.81). As the salinity level increased, the number of leaves decreased (**Table 4**).

**Table 1:** Root colonization of *D. kaki* seedlings inoculated with different mycorrhizal species under different salinity levels.

Mycorrhizal species	NaCl concentration	Root colonization (%)
*G. clarium*	0 mM	71.0
	50 mM	62.0
	75 mM	56.5
	100 mM	43.0
	150 mM	29.5
*E. etunicatum*	0 mM	68.5
	50 mM	66.5
	75 mM	45.5
	100 mM	37.0
	150 mM	32.0

**Table 2:** Results of a two-way analysis of variance (ANOVA) of inoculation treatments (AMF) and salinity treatment (Dose) effects and their interaction (AMF x Dose) for the dependent variables considered based on randomized complete block design (Block + AMF + Dose + AMF x Dose).

Trait	tAMF (*F*)	AMF (*p*)	Dose (*F*)	Dose (*p*)	AMF x Dose (*F*)	AMF x Dose (*p*)	Block (*F*)	Block *(p*)	CV%	*R* ^2^
Number of Leaves	42.68	0.0000	6.44	0.0001	4.15	0.0003	0.50	0.8057	24.31	0.64
Fresh Root Weight(g)	66.60	0.0000	2.77	0.0329	1.46	0.1861	1.91	0.0891	36.26	0.68
Fresh Stem Weight(g)	51.84	0.0000	1.96	0.1088	0.40	0.9184	1.72	0.1259	40.81	0.61
Dry Root Weight(g)	36.05	0.0000	2.70	0.0361	1.33	0.2392	1.86	0.0973	40.04	0.57
Dry Stem Weight(g)	36.37	0.0000	2.51	0.0483	0.37	0.9349	0.86	0.5260	49.35	0.53
SPAD	22.60	0.0000	10.87	0.0000	1.80	0.0890	1.00	0.4314	11.82	0.57
PSII	63.20	0.0000	5.48	0.0006	1.68	0.1158	1.18	0.3250	14.93	0.70
Leaf Symptoms Score	6.97	0.0016	63.73	0.0000	3.79	0.0008	0.24	0.9623	29.28	0.78
Leaf Cl Conc.(%)	51.68	0.0000	457.11	0.0000	10.01	0.0000	0.90	0.4958	8.04	0.96
Root Cl Conc.(%)	3.67	0.0297	19.17	0.0000	2.17	0.0382	0.77	0.5924	19.94	0.56
Leaf Ca Conc.(%)	0.44	0.6487	2.90	0.0266	4.76	0.0001	0.12	0.9931	25.18	0.38
Leaf K Conc.(%)	38.35	0.0000	2.61	0.0411	2.21	0.0348	1.75	0.1205	28.36	0.58
Leaf Na Conc.(%)	19.87	0.0000	19.55	0.0000	2.84	0.0077	0.72	0.6366	42.30	0.63
Leaf P Conc.(%)	52.15	0.0000	4.28	0.0034	1.13	0.3502	0.99	0.4385	28.70	0.62
Leaf Zn Conc.(ppm)	3.77	0.0271	4.19	0.0038	3.41	0.0019	1.01	0.4244	30.98	0.41
Root Ca Conc.(%)	9.87	0.0001	4.68	0.0018	3.26	0.0028	0.57	0.7563	28.45	0.45
Root K Conc.(%)	6.06	0.0035	9.76	0.0000	3.64	0.0011	1.90	0.0901	29.26	0.52
Root Na Conc.(%)	8.12	0.0006	29.16	0.0000	4.14	0.0003	1.36	0.2387	26.64	0.68
Root P Conc.(%)	167.85	0.0000	7.56	0.0000	2.15	0.0398	1.08	0.3840	24.37	0.82
Root Zn Conc.(ppm)	155.89	0.0000	15.05	0.0000	4.77	0.0001	2.02	0.0719	29.40	0.83

CV(%), Coefficient of Variation.

R² (R-squared), Represents how much of the total variation in that trait is explained by the model Block + AMF + Dose + AMF×Dose combined.

**Table 3:** Root fresh weight (g), stem fresh weight (g), Root dry weight (g) and Stem dry weight (g) of AMF inoculated *D. kaki* under salinity stress.

AMF treatments	NaCl level	Root fresh weight (g)	Stem fresh weight (g)	Root dry weight (g)	Stem dry weight (g)
*G. clarium*		12.66^a^	6.25^a^	2.60^a^	1.54^a^
*E. etunicatum*		14.25^a^	7.14^a^	2.89^a^	1.61^a^
Control		4.47^b^	2.26^b^	1.19^b^	0.50^b^
	0 mM	12.82^a^	6.13	2.82^a^	1.49^a^
	50 mM	11.03^ab^	5.42	2.40^ab^	1.37^ab^
	75 mM	9.83^b^	5.14	2.16^bc^	1.20^abc^
	100 mM	9.65^b^	5.03	1.96^bc^	1.11^bc^
	150 mM	8.96^b^	4.20	1.70^c^	0.92^c^
*G. clarium*	0 mM	17.45	7.78	3.43	1.77
	50 mM	12.29	6.30	2.50	1.65
	75 mM	11.87	6.21	2.45	1.62
	100 mM	10.20	5.94	2.33	1.50
	150 mM	11.50	4.99	2.18	1.21
*E. etunicatum*	0 mM	16.06	7.81	3.42	2.00
	50 mM	16.00	7.78	3.30	1.81
	75 mM	13.05	7.09	2.93	1.47
	100 mM	14.50	6.99	2.65	1.42
	150 mM	11.64	6.02	2.15	1.32
Control	0 mM	4.97	2.80	1.60	0.70
	50 mM	4.80	2.18	1.40	0.67
	75 mM	4.57	2.59	1.10	0.51
	100 mM	4.26	2.15	1.06	0.41
	150 mM	3.75	1.59	0.78	0.24
*LSDamf*		*1.820*	*1.030*	*0.434*	*0.282*
*LSDsalinity*		*2.350*	*-*	*0.560*	*0.364*
*LSDamf*×*salinity*		*-*	*-*	*-*	*-*

Differences between the means were shown with different letters within the same column.

**Table 4:** Leaf number, symptom score, leaf Chl concentration (SPAD readings) and chlorophyll fluorescence (*Fv*’/*Fm’*) of AMF inoculated *D. kaki* under salinity stress.

AMF treatments	NaCl level	Leaf number	Symptom score	SPAD	*Fv’*/*Fm’*
*G. clarium*		11.94^a^	2.17^b^	28.09^b^	0.72^a^
*E. etunicatum*		11.31^a^	1.91^b^	33.01^a^	0.71^a^
Control		6.97^b^	2.49^a^	33.51^a^	0.48^b^
	0 mM	12.10^a^	0.33^d^	35.23^a^	0.72^a^
	50 mM	11.10^a^	2.10^c^	32.23^b^	0.64^b^
	75 mM	10.81^a^	2.52^b^	32.39^b^	0.63^b^
	100 mM	8.88^b^	2.76^b^	28.96^c^	0.62^bc^
	150 mM	7.50^b^	3.24^a^	28.84^c^	0.55^c^
*G. clarium*	0 mM	14.14^a^	1.00^f^	38.33	0.75
	50 mM	12.43^ab^	2.00^de^	34.04	0.71
	75 mM	11.86^abc^	2.29^cd^	35.87	0.74
	100 mM	11.43^bcd^	2.29^cd^	28.67	0.71
	150 mM	9.86^c-f^	3.29^a^	30.61	0.66
*E. etunicatum*	0 mM	11.71^abc^	0.00^g^	34.14	0.75
	50 mM	12.57^ab^	1.43^ef^	32.93	0.72
	75 mM	12.57^ab^	2.43^cd^	34.31	0.69
	100 mM	10.71^b-e^	2.57^bcd^	32.13	0.74
	150 mM	9.00^def^	3.14^ab^	31.51	0.66
Control	0 mM	10.43^b-f^	0.00^g^	33.23	0.65
	50 mM	8.29^ef^	2.86^abc^	29.73	0.46
	75 mM	8.00^f^	2.86^abc^	26.99	0.46
	100 mM	4.50^g^	3.43^a^	26.09	0.41
	150 mM	3.66^g^	3.29^a^	24.40	0.40
*LSDamf*		*1.144*	*0.296*	*1.769*	*0.043*
*LSDsalinity*		*1.477*	*0.383*	*2.285*	*0.056*
*LSDamf*×*salinity*		*1.476*	*0.663*	*-*	*-*

Differences between the means were shown with different letters within the same column.

### 3.2 Leaf chlorophyll concentration, fluorescence and salinity symptoms

Leaf chlorophyll concentrations were significantly affected by inoculation treatments according to a two-way ANOVA (**Table 2**). Plants inoculated with *G. clarium* (33.51) and non-inoculated plants (33.01) had higher chlorophyll concentrations than plants inoculated with *E. etunicatum* (28.09). Additionally, different salinity levels significantly affected the chlorophyll concentration in the leaves of *D. kaki* seedlings. The highest chlorophyll concentration was observed in control plants (35.23), whereas it was the lowest in plants treated with 100 mM (28.96) and 150 mM (28.84) NaCl. On the other hand, the interaction effect of mycorrhiza × salt treatment was statistically insignificant. Chlorophyll fluorescence in the light-adapted stage (*Fv’*/*Fm’*) of *D. kaki* leaves was significantly affected by the main effects of AMF inoculation and salinity treatments. PSII efficiency in the leaves of plants inoculated with *E. etunicatum* (0.71) and *G. clarium* (0.72) was higher than that of the control plants (0.48). Besides, different salinity levels significantly affected (*Fv’*/*Fm’*) of *D. kaki* leaves, according to a two-way ANOVA conducted (**Table 4**). The highest chlorophyll fluorescence was determined in control (0.72), whereas it was the lowest in 100 mM (0.62) and 150 mM (0.55) NaCl-treated plants. Mycorrhiza × salt treatment interaction effect was not statistically significant in terms of chlorophyll fluorescence of *D.kaki* leaves at the light-adapted stage. AMF inoculation treatments significantly affected the leaf salinity symptom scores of *D. kaki*. *E. etunicatum* and *G. clarium* inoculated plants had lower symptom scores in comparison to non-inoculated plants, 1.91 and 2.17, respectively (**Table 4**). Leaves of plants without mycorrhiza inoculation were observed as leaf blight-wilt subjectively (2.49). Similarly, salt doses and AMF × salt treatment interaction effects were statistically significant. The highest symptom score was observed in plants treated with 150 mM NaCl (3.24), whereas symptoms were lowest in control plants (0 mM NaCl). Regarding the AMF × salt treatment interaction, control plants had healthy leaf color, whereas leaf burns and wilting were observed at increasing NaCl levels (**Table 4**).

### 3.3 Leaf mineral composition

Both main effects and their interactions were statistically significant on leaf Cl concentrations of *D. kaki*. Control plants without mycorrhiza treatment had a higher leaf Cl concentration (113.89 ppm) than *E. etunicatum* (95.83 ppm) and *G. clarium* (97.41 ppm; **Table 5**). In terms of salinity treatments, the lowest leaf Cl concentration was observed in plants treated with 0 mM NaCl (41.95 ppm). Generally, leaves accumulated more Cl ions as the NaCl dose increased. Regarding the AMF × salt treatment interaction, the lowest Cl concentration was observed in the leaves of plants inoculated with *E. etunicatum* under 0 mM NaCl (32.00 ppm). Leaves of plants treated with *G. clarium* had lower Cl concentrations at 50, 75, and 100 mM NaCl than those inoculated with *E. etunicatum*. *G. clarium* treated plants accumulated 71.00 ppm, 105.14 ppm, and 128.86 ppm Cl in 50, 75, and 100 mM NaCl, respectively (**Table 5**). On the other hand, plants inoculated with *G. etinucatum* accumulated 82.66 ppm, 111.63 ppm, and 134.86 ppm Cl, respectively. In the leaves of 150 mM NaCl and non-inoculated plants, 143.29 ppm Cl was determined. Leaves of plants inoculated with *E. etunicatum* and treated with 150 mM NaCl accumulated 118.00 ppm Cl ion. The main effects of AMF and salinity treatments significantly affected the leaf Ca concentrations. In terms of salinity levels, the highest leaf Ca concentration was observed in control (2.00%), whereas it was the lowest in the 75 mM (1.62%) and 150 mM (1.61%) NaCl treatments. In terms of AMF × salt treatment interaction, the highest leaf Ca concentration was determined in *G. clarium* inoculated and 50 mM NaCl treated plants (2.50%). Both main and interaction effects were significant on leaf K concentrations of *D. kaki* (**Table 5**). Regarding AMF inoculation treatments, the highest leaf K concentration was observed in the control plants (2.04%), followed by *E. etunicatum* (1.43%), and the lowest in plants inoculated with *G. clarium* (1.15%). In terms of the interaction effect, the highest K concentration was obtained from the leaves non-inoculated and 150 mM NaCl treatment (2.56%), whereas it was the lowest in the leaves of *G. clarium* inoculated and 150 mM NaCl (0.99%) treated plants. Na concentration of the leaves of *D. kaki* was significantly affected by the AMF and salinity treatments, but not their interaction effect. Based on the main effect of AMF treatments, the highest leaf Na concentration was determined in control plants (0.58%), whereas it was determined as 0.34% in *G. clarium* inoculated plants and 0.35% in *E. etunicatum* inoculated ones. In terms of salinity treatments, the highest leaf Na concentration was determined in 100 mM NaCl-treated plants (0.61%) and the lowest in control (0.14%). Besides, leaf Na concentrations were found to be like the other treatments. In terms of AMF × salt treatment interaction, the highest leaf Na concentration was determined as 0.89% in 100 mM NaCl application of non-AMF treated control plants, whereas the lowest leaf Na concentration was determined as 0.11% in non-salt treated of inoculated plants. AMF inoculation and salt treatments were statistically significant, while their interaction was not significantly different (**Table 5**). The highest leaf P concentration was determined in *E. etunicatum* (0.16%) and *G. clarium* (0.15%) inoculated plants, and the lowest P concentration was in control (0.08%). In terms of salinity treatment effects, the highest leaf P concentration was observed with 50 mM NaCl and 0 mM NaCl applications. In terms of leaf Zn concentration, both the main and the interaction effects were significant. The highest leaf Zn concentration was determined as 22.75 ppm in *G. clarium* and 22.59 ppm in *E. etunicatum*, whereas it was the lowest in the control (18.90 ppm). Regarding the salinity main effect, leaf Zn concentration was highest in control plants (24.34 ppm) and lowest in 75 mM NaCl-treated plants (17.14 ppm). According to the interaction effect, the highest leaf Zn concentration was in *G. clarium* 50 mM NaCl (30.03 ppm), *G. clarium* 0 mM NaCl (28.03 ppm) and control 100 mM NaCl (27.54 ppm) treatments (**Table 5**).

**Table 5:** Leaf Cl (mg l-1), Ca (%), K(%), Na (%), P(%) and Zn (ppm) concentrations of AMF inoculated *D. kaki* under salinity stress.

AMF treatments	NaCl level	Cl (mg/g)	Ca (%)	K (%)	Na (%)	P (%)	Zn (ppm)
*G. clarium*		97.41^b^	1.81	1.15^c^	0.34^b^	0.15^a^	22.75^a^
*E. etunicatum*		95.83^b^	1.72	1.43^b^	0.35^b^	0.16^a^	22.59^a^
Control		113.89^a^	1.80	2.04^a^	0.58^a^	0.08^b^	18.90^b^
	0 mM	41.95^d^	2.00^a^	1.38^b^	0.14^c^	0.14^a^	24.34^a^
	50 mM	88.05^c^	1.87^ab^	1.39^b^	0.46^b^	0.14^a^	23.52^ab^
	75 mM	116.35^b^	1.62^b^	1.51^ab^	0.44^b^	0.10^c^	17.14^c^
	100 mM	132.29^a^	1.78^ab^	1.70^a^	0.61^a^	0.13^ab^	22.37^ab^
	150 mM	133.24^a^	1.61^b^	1.69^a^	0.48^b^	0.12^bc^	19.70^bc^
*G. clarium*	0 mM	43.61^h^	2.09^abc^	1.05^fg^	0.11^g^	0.14	28.03^a^
	50 mM	71.00^g^	2.50^a^	1.27^efg^	0.44^b-e^	0.18	30.03^a^
	75 mM	105.14^e^	1.69^b-e^	1.19^efg^	0.36^def^	0.12	16.06^d^
	100 mM	128.86^c^	1.57^def^	1.22^efg^	0.54^bcd^	0.15	19.66^bcd^
	150 mM	138.43^ab^	1.21^f^	0.99^g^	0.26^efg^	0.15	20.00^bcd^
*E. etunicatum*	0 mM	32.00^i^	1.96^bcd^	1.34^d-g^	0.11^g^	0.19	26.29^ab^
	50 mM	82.66^f^	1.66^c-f^	1.33^d-g^	0.32^ef^	0.17	23.76^abc^
	75 mM	111.63^de^	1.64^c-f^	1.43^d-g^	0.37^def^	0.13	19.23^cd^
	100 mM	134.86^abc^	1.63^c-f^	1.52^cde^	0.40^cde^	0.15	19.91^bcd^
	150 mM	118.00^d^	1.71^b-e^	1.50^c-f^	0.57^bc^	0.15	23.74^abc^
Control	0 mM	50.23^h^	1.95^bcd^	1.75^cd^	0.19^fg^	0.09	18.71^cd^
	50 mM	110.50^de^	1.45^ef^	1.57^cde^	0.62^b^	0.09	16.77^cd^
	75 mM	132.29^bc^	1.51^def^	1.97^bc^	0.60^b^	0.07	16.14^d^
	100 mM	133.14^bc^	2.14^ab^	2.35^ab^	0.89^a^	0.08	27.54^a^
	150 mM	143.29^a^	1.93^bcd^	2.56^a^	0.60^b^	0.05	15.34^d^
*LSDamf*		*3.896*	*-*	*0.210*	*0.084*	*0.017*	*3.151*
*LSDsalinity*		*5.030*	*0.266*	*0.272*	*0.109*	*0.022*	*4.068*
*LSDamf*×*salinity*		*8.712*	*0.461*	*0.471*	*0.189*	*-*	*7.046*

Differences between the means were shown with different letters within the same column.

### 3.4 Root mineral composition

Both main effects and their interactions were statistically significant for leaf Cl concentrations in *D. kaki* (**Table 2**). Root Cl concentration was higher in mycorrhiza-inoculated plants (*E. etunicatum*: 46.81 ppm, *G. clarium*: 46.57 ppm) in comparison to the control (41.66 ppm). Comparing the main effect of salt treatments, the highest root Cl concentrations were observed in the 50 mM (51.14 ppm) and 100 mM (50.44 ppm) NaCl treatments, whereas the lowest was in the control (30.50 ppm). In terms of AMF × salt treatment interaction, the highest root Cl concentration was detected in *E. etunicatum* inoculated and 50 mM NaCl (57.29 ppm) treated plants, whereas it was the lowest in 0 mM NaCl (24.71 ppm) treatment without mycorrhizae (**Table 6**). A two-way ANOVA indicated a significant main effect of AMF inoculation and salinity treatments and also their interaction (*p ≤ 0.01*) on the root Ca concentrations. NaCl treatments resulted in increased root Ca concentrations in both *E. etunicatum* (0.78%) and *G. clarium* (0.66%). The highest root Ca concentration across salinity levels was observed in plants treated with 100 mM NaCl (0.79%) and 150 mM NaCl (0.72%). In terms of AMF × salt treatment interaction, the highest root Ca concentration was determined in *E. etunicatum* treated with 100 mM NaCl (1.06%), and the lowest in 50 mM NaCl (0.44%) treatment without AMF (**Table 6**). Both main and interaction effects were significant on root K concentration. The highest root K concentration was detected in the control (0.68%) and *E. etunicatum* (0.65%) treatments, whereas it was the lowest in *G. clarium*-inoculated plants (0.54%). In terms of the AMF × salt treatment interaction effect, the highest root K concentration was determined in *E. etunicatum* inoculated and 150 mM NaCl-treated plants (0.91%), whereas it was the lowest in non-inoculated and 0 mM NaCl-treated samples (0.39%) treated samples (**Table 6**). The main effects and their interaction were statistically significant for root Na concentration in *D. kaki*. In terms of mycorrhiza treatments, the highest root Na concentration was determined in *E. etunicatum* plants (0.36%), while it was determined as 0.31% in *G. clarium* and 0.28% in control. Regarding salinity treatments, the highest root Na concentration was determined in the 100 mM NaCl treatment (0.42%) and the lowest in the control (0.16%). The highest root Na concentration was determined as 0.48% in both *E. etunicatum* and *G. clarium* 100 mM NaCl treatments, while the lowest leaf Na concentration was found in non-AMF-inoculated and 0 mM NaCl treatment (0.12%) plants, regarding the AMF × salt treatment interaction (**Table 6**). AMF inoculation, salinity treatments, and their interaction were statistically significant for root P concentrations, as determined by a two-way ANOVA (**Table 2**). The highest root P concentration was observed in *E. etunicatum* (0.21%) and *G. clarium* (0.19%) inoculations, whereas it was the lowest in the control (0.06%). In terms of salinity treatments, the highest root P concentration was 0.17% in both 100 mM and 150 mM NaCl treatments. The lowest root P concentration was observed at 0.12% NaCl application. AMF, salinity, and their interaction were statistically significant for root Zn concentrations (**Table 2**). In mycorrhiza treatments, the highest root Zn concentration was determined as 59.85 ppm in *G. clarium* and 66.02 ppm in *E. etunicatum* inoculated plants, whereas it was the lowest in the control (13.35 ppm). Root Zn concentration was highest in 50 mM NaCl plants (64.88 ppm) and lowest in 75 mM NaCl (38.03 ppm) and 150 mM NaCl (38.03 ppm) treatments regarding salinity. Considering the AMF × salt treatment interaction, the highest root Zn concentration was determined in *E. etunicatum* inoculated and 50 mM NaCl (92.53 ppm) treated plants, together with *G. clarium* inoculated and 50 mM NaCl (86.23 ppm) treated plants. The lowest root Zn concentration was determined in 0 mM NaCl (9.29 ppm) treated plants without mycorrhiza treatment, followed by 50 mM NaCl (15.89 ppm), 75 mM NaCl (11.46 ppm), 100 mM NaCl (17.57 ppm), and 150 mM NaCl (12.57 ppm) treatments (**Table 4**).

**Table 6:** Root Cl (mg l^-1^), Ca (%), K(%), Na (%), P(%) and Zn (ppm) concentrations of AMF inoculated *D. kaki* under salinity stress.

AMF treatments	NaCl level	Cl (mg/g)	Ca (%)	K (%)	Na (%)	P (%)	Zn (ppm)
*G. clarium*		46.57^b^	0.66^b^	0.54^b^	0.31^b^	0.19^a^	59.85^a^
*E. etunicatum*		46.81^a^	0.78^a^	0.65^a^	0.36^a^	0.21^a^	66.02^a^
Control		41.66^b^	0.58^b^	0.68^a^	0.28^b^	0.06^b^	13.35^b^
	0 mM	30.50^c^	0.59^bc^	0.46^b^	0.16^d^	0.15^b^	42.55^bc^
	50 mM	51.14^a^	0.57^c^	0.68^a^	0.34^bc^	0.16^ab^	64.88^a^
	75 mM	44.04^b^	0.69^ab^	0.52^b^	0.31^c^	0.12^c^	36.68^c^
	100 mM	50.44^a^	0.79^a^	0.71^a^	0.42^a^	0.17^a^	49.91^b^
	150 mM	48.95^ab^	0.72^a^	0.75^a^	0.37^ab^	0.17^a^	38.03^c^
*G. clarium*	0 mM	30.29^fg^	0.68^bcd^	0.49^e-h^	0.19^efg^	0.20^b^	61.13^b^
	50 mM	57.29^a^	0.68^bcd^	0.67^b-e^	0.33^cd^	0.21^b^	86.23^a^
	75 mM	49.83^abc^	0.68^bcd^	0.43^gh^	0.28^de^	0.14^c^	44.71^c^
	100 mM	47.29^bcd^	0.57^cde^	0.58^c-h^	^0.40abc^	0.20^b^	51.43^bc^
	150 mM	48.14^a-d^	0.68^bcd^	0.52^d-h^	0.35^bcd^	0.22^ab^	55.77^bc^
*E. etunicatum*	0 mM	36.50^ef^	0.57^cde^	0.50^d-h^	0.16^fg^	0.20^b^	57.23^bc^
	50 mM	49.00^abc^	0.60^cde^	0.76^abc^	0.48^a^	0.21^b^	92.53^a^
	75 mM	39.00^def^	0.87^ab^	0.45^fgh^	0.28^de^	0.16^c^	53.86^bc^
	100 mM	54.29^ab^	1.06^a^	0.65^b-f^	0.48^a^	0.23^ab^	80.73^a^
	150 mM	55.29^ab^	0.80^b^	0.91^a^	0.43^ab^	0.25^a^	45.74^c^
Control	0 mM	24.71^g^	0.53^de^	0.39^h^	0.12^g^	0.05^d^	9.29^d^
	50 mM	47.14^bcd^	0.44^e^	0.62^b-g^	0.22^ef^	0.07^d^	15.89^d^
	75 mM	43.29^cde^	0.53^de^	0.69^bcd^	0.36^bcd^	0.05^d^	11.46^d^
	100 mM	49.74^abc^	0.73^bc^	0.89^a^	0.38^bc^	0.08^d^	17.57^d^
	150 mM	43.43^cde^	0.67^bcd^	0.81^ab^	0.34^cd^	0.05^d^	12.57^d^
*LSDamf*		*4.229*	*0.089*	*0.089*	*0.041*	*0.018*	*6.695*
*LSDsalinity*		*5.460*	*0.115*	*0.115*	*0.053*	*0.023*	*8.644*
*LSDamf*×*salinity*		*9.458*	*0.200*	*0.199*	*0.091*	*0.040*	*14.971*

Differences between the means were shown with different letters within the same column.

### 3.5 Correlation and regression analyses

Significant correlations between investigated parameters were determined, except for plant growth parameters (plant height, number of leaves, fresh root weight, fresh stem weight, dry root weight, and dry stem weight) versus leaf symptoms and both leaf and root Ca and Cl concentrations (**Figure 1**). Except for the correlations between plant growth parameters, the correlation coefficients between Chl content and PSII (0.84), SPAD and leaf P concentration (0.79) were very significant. Also, a significant correlation was observed between leaf and root P concentrations (r = 0.91). Leaf Zn and Ca concentrations were highly correlated (r = 0.68). Therefore, regression analyses were performed on these correlations to predict plant growth using highly correlated variables under different AMF inoculations. **Figure 2** presented the prediction (not validated-associative) of total dry weight based on leaf Chl concentration, PSII, leaf P, and Zn concentration. Significant predictions were determined in control and *G. clarium* plants (*p=* 0.038 and *p=* 0.024, respectively). Regression between measured total dry weight (g) and associative total dry weight (g) calculated based on leaf Cl, Ca, Zn and P concentrations of *D. kaki* inoculated with different AMF were presented in **Figure 3**. The prediction of total dry weight was significant in plants inoculated with *G. clarium* (*p* = 0.003). Considering the prediction of total plant dry weight based on root Cl, Ca, Zn, and P concentrations in **Figure 4**, both *G. clarium* and *E. etunicatum* were significant (*p* = 0.011 and *p* = 0.016, respectively).

**Figure 1:**
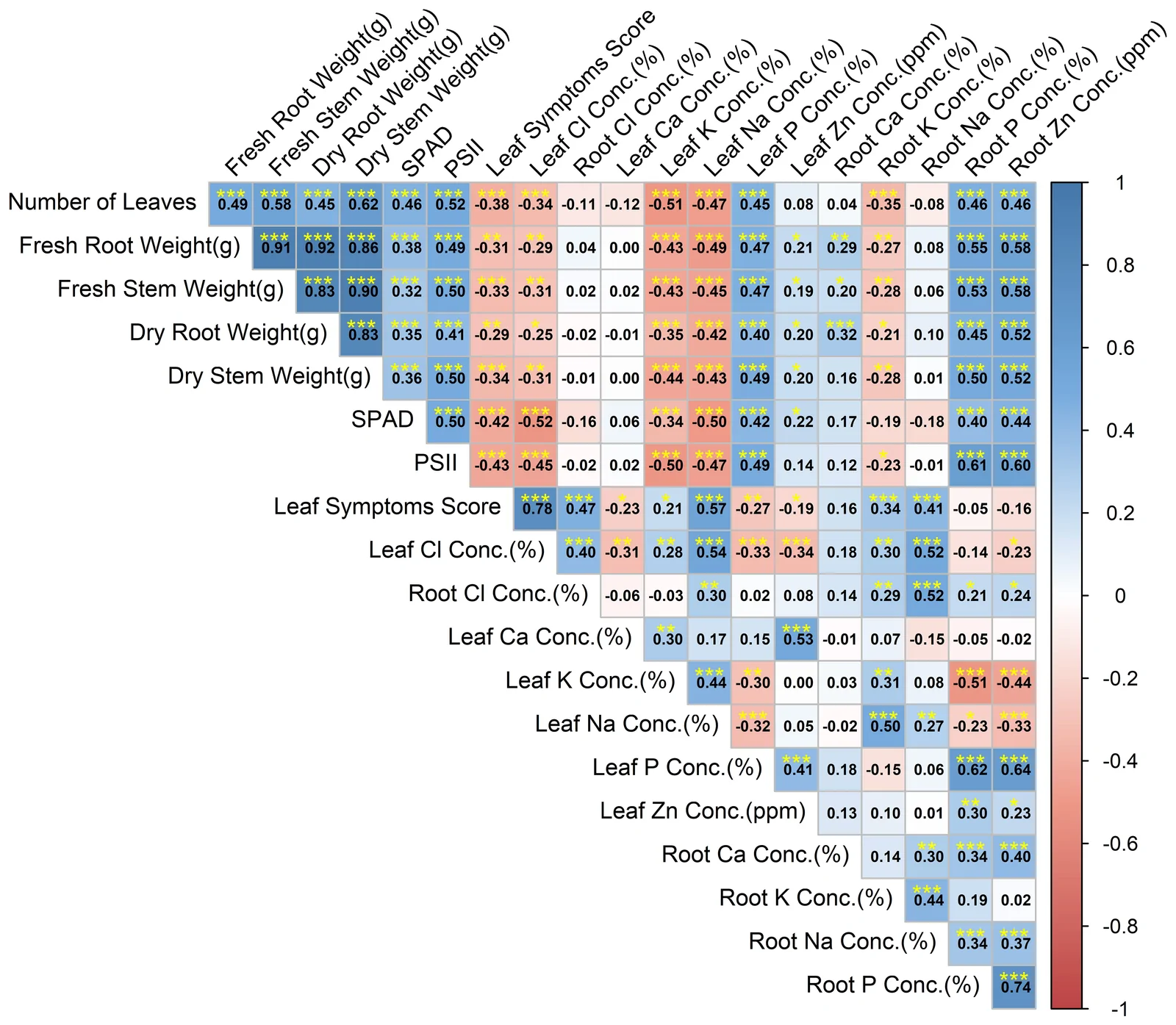
Correlation coefficient analysis between investigated variables.

**Figure 2:**
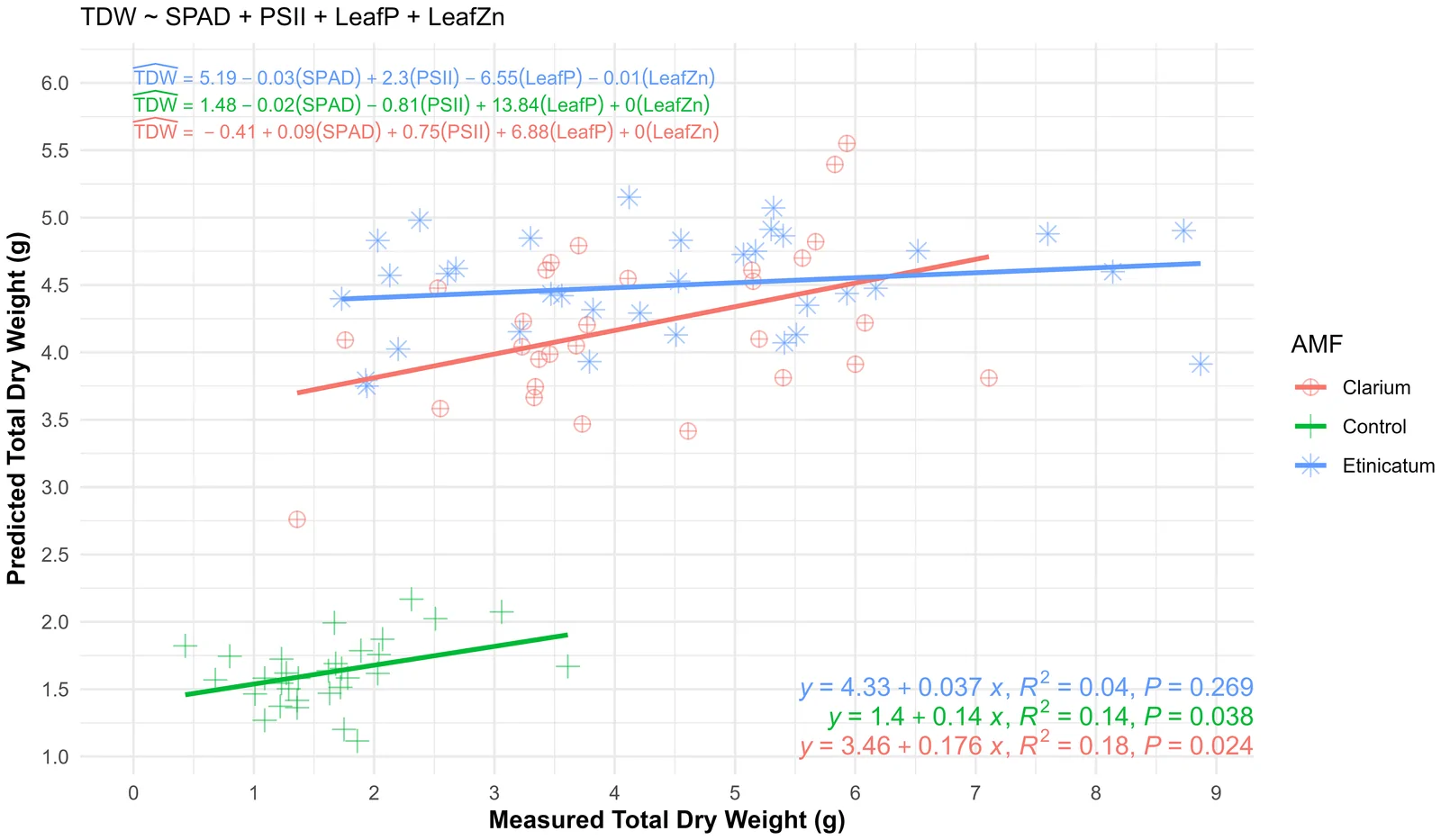
Regressions between measured total dry weight (g) and associative/explanatory total dry weight (g) calculated based on leaf chlorophyll concentration, chlorophyll fluorescence leaf P and Zn concentrations of *D. kaki* inoculated with different AMF.

**Figure 3:**
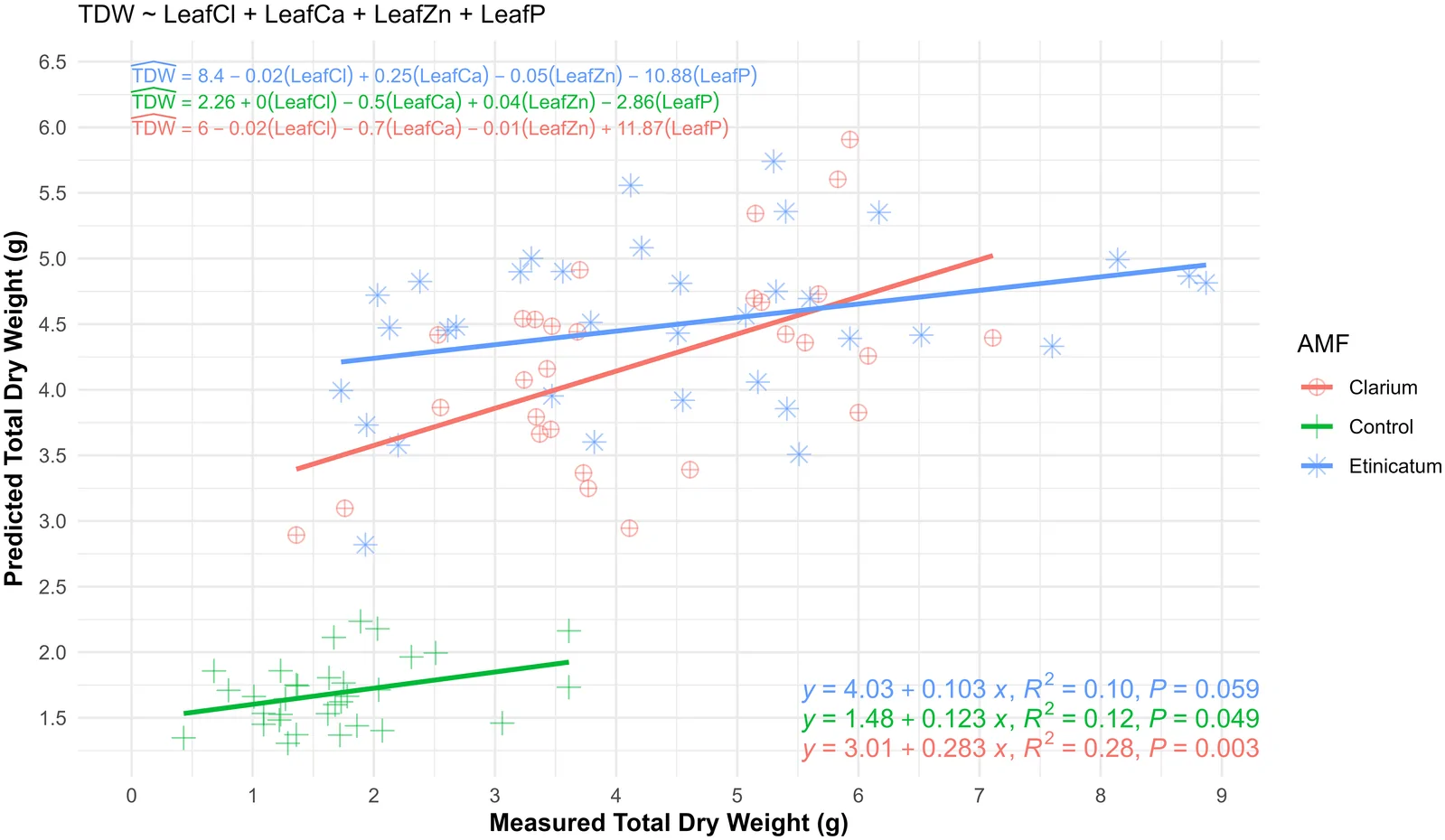
Regressions between measured total dry weight (g) and associative/explanatory total dry weight (g) calculated based on leaf Cl, Ca, Zn and P concentrations of *D. kaki* inoculated with different AMF.

**Figure 4:**
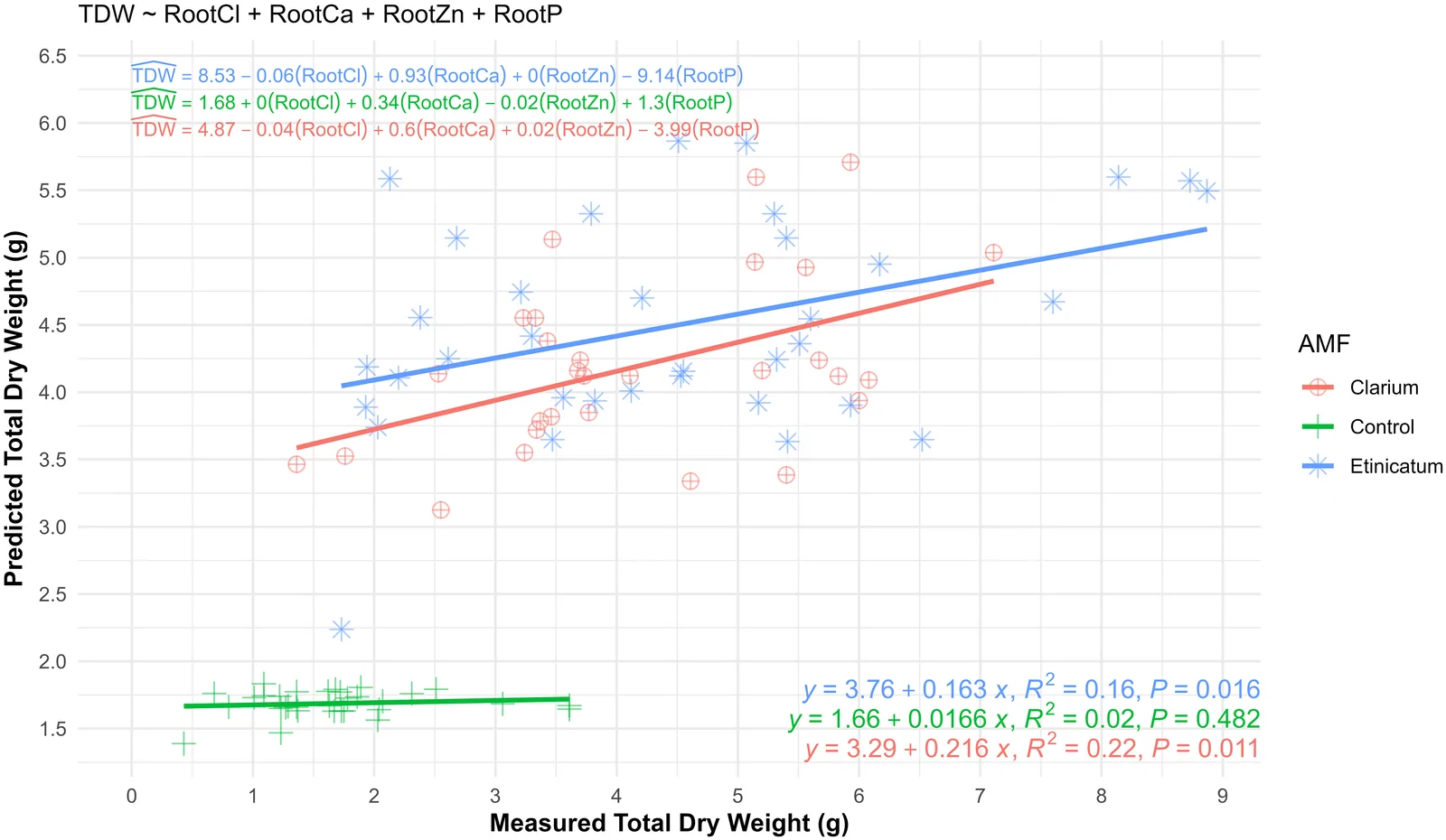
Regressions between measured total dry weight (g) and associative/explanatory total dry weight (g) calculated based on root Cl, Ca, Zn and P concentrations of *D. kaki* inoculated with different AMF.

## 4 Discussion

This study investigated the effects of salinity stress on growth, physiological parameters, and tissue nutrient concentration in *D. kaki* seedlings, with and without AMF inoculation. The results demonstrate that mycorrhizal colonization, particularly with *E. etunicatum* and *G. clarium*, significantly improved seedling performance under saline conditions relative to non-inoculated controls. These findings align with previous reports documenting the positive effects of AMF on growth and development in various cultivated species, including *D. kaki* (Oyetunji et al., 2007; Hajiboland et al., 2010; Ortas et al., 2011; Wu et al., 2011; Incesu et al., 2015; Machineski et al., 2018; Keller-Pearson et al., 2020; Tang et al., 2023).

In our study increasing salinity levels resulted as a decrease in terms of AMF colonization. It was previously determined that AMF are able to adapt to salt concentrations ranging from 0 to 50 mM at moderate levels and even enhance plant growth under such conditions. However, high salt stress appears to inhibit infection development, albeit with a low slope. Similarly, Asma et al. (2020) reported a negative correlation between mycorrhizal infection intensity and salt concentration. In general, it has been stated that under high salt-dose conditions, hyphal development is inhibited, spore viability and spore density decrease, and this leads to a reduction in mycorrhizal hyphal biomass (Juniper and Abbott, 2006). Furthermore, Boorboori and Lackoova (2025) reported that Na^+^ ions exert a direct toxic effect on AMF functions, thereby reducing colonization rates.

Consistent with established literature, increasing salinity levels led to a progressive decline in seedling growth and development. This adverse effect of salinity on morphological traits—including plant weight, leaf dimensions, and root length—has been well documented and ultimately contributes to reductions in crop yield (Pandit et al., 2024). Biomass accumulation is widely recognized as a key indicator of plant salt tolerance (Da Silva et al., 2008; Hajiboland et al., 2010; Navarro et al., 2024). In the present study, mycorrhizal inoculation, particularly with *E. etunicatum*, conferred enhanced salt tolerance in *D. kaki*, with notable effects observed under 50 mM NaCl. While these findings were obtained using seed-propagated plants, further validation using vegetatively propagated material is recommended to confirm the efficacy of the persimmon–*E. etunicatum* combination under saline conditions.

Physiological assessments revealed that chlorophyll content, measured via SPAD readings, was positively influenced by mycorrhizal colonization and declined with increasing salinity. Reduced chlorophyll content in non-mycorrhizal plants under salt stress has been previously reported in tomato (Hajiboland et al., 2010), peanut (Al-Khaliel, 2010), *D. virginiana* (Incesu et al., 2015), and maize (Zare-Maivan et al., 2017). Conversely, mycorrhizal inoculation has been shown to enhance photosynthetic rates, gas exchange parameters, chlorophyll content, and water use efficiency under saline conditions, as demonstrated in *Ocimum basilicum* (Elhindi et al., 2017). In agreement with the findings of Navarro et al. (Navarro et al., 2024) in citrus, the present study confirms that AMF colonization significantly improves physiological performance under salinity stress, as evidenced by enhanced chlorophyll content and photosystem II (PSII) photochemical efficiency in mycorrhizal-treated persimmon plants. Salinity, in particular, impairs the oxygen-evolving complex (OEC) and suppresses the PSII reaction center by degrading the D1 protein on the acceptor side, thereby slowing electron transfer. The untransferred electrons subsequently react with free oxygen, generating reactive oxygen species (ROS) that cause damage to PSII (Chen et al., 2020). On the other hand, our findings described only in terms of the associations between AMF inoculation, salinity, growth, and mineral/physiological parameters.

The use of beneficial soil microorganisms, including AMF, is a promising strategy to increase plant tolerance to salinity. The mechanisms by which AMF enhance host plant flexibility under salt-stressed conditions include facilitating water and tissue nutrient concentration, enhancing photosynthetic capacity, modulating antioxidant responses, inhibiting excessive ion accumulation, and promoting root and shoot biomass (Boorboori and Lackóová, 2025). Soil salinity is attributed to various salts, with sodium chloride being the most prevalent in arid and semi-arid regions (Flowers et al., 1977). Plant responses to salinity vary, with some species exhibiting greater sensitivity to chloride toxicity and others to sodium toxicity. Incesu et al. (2015) reported that *D. kaki* accumulates more sodium than *D. virginiana*, whereas the latter accumulates more chloride. In the current study, both AMF species demonstrated comparable effects on ion regulation, significantly reducing sodium and chloride accumulation relative to non-inoculated controls. This consistent reduction in toxic ion uptake underscores the potential of these mycorrhizal associations to improve persimmon salt tolerance in affected soils.

AMF are known to enhance the acquisition of phosphorus (P) and zinc (Zn) by host roots (Moshfeghi et al., 2019; Nguyen et al., 2019; Saboor et al., 2021). Improved phosphorus nutrition can indirectly contribute to salt tolerance by supporting ATP production, membrane stability, and antioxidant metabolism, thereby increasing the plant’s resistance to osmotic stress. Phosphorus is essential throughout all vegetative and reproductive stages, while zinc, though required in minute quantities, positively influences yield, fruit set, and fruit quality (Rafiullah et al., 2020; Saboor et al., 2021; Abdi et al., 2023). Despite the antagonistic interaction between P and Zn, AMF can regulate the uptake and accumulation of both nutrients (Saboor et al., 2021). Ortas (2012) similarly reported increased P and Zn content in mycorrhizal-inoculated citrus plants, alongside enhanced growth and uptake of K, Fe, and Cu. Salinity influences plant P concentration in many crops (Poss et al., 1985), and selecting efficient AMF strains may improve tolerance to soil salinity. In the present study, leaf sodium and potassium concentrations were higher in control plants than in mycorrhizal-treated plants, whereas leaf P and Zn concentrations were higher in mycorrhizal-treated plants. Notably, the approximately two-fold increase in P concentration observed in mycorrhizal plants compared to controls represents a significant advantage for phosphorus fertilization requirements in cultivation. Improved P nutrition can also indirectly contribute to salt tolerance by supporting ATP production, membrane stability, and antioxidant metabolism, thereby increasing the plant’s resistance to osmotic stress (Khan et al., 2023).

Root potassium and zinc concentrations were higher in both *G. clarium* and *E. etunicatum* treatments relative to controls. Potassium (K^+^) is a critical macronutrient involved in numerous physiological processes, including regulation of membrane potential, enzyme activation, and responses to biotic and abiotic stresses (Kafle et al., 2022). Previous studies have reported positive effects of AMF on potassium acquisition in colonized plants (Estrada et al., 2013; Garcia et al., 2017; Ye et al., 2017; Liu et al., 2019), with Kafle et al. (Kafle et al., 2022) demonstrating the existence of a mycorrhizal uptake pathway for K^+^ nutrition in *Medicago truncatula*. In the present study, root potassium accumulation was lower in *G. clarium*-inoculated plants but higher in *E. etunicatum*-inoculated plants. Similarly, root concentrations of calcium, sodium, phosphorus, and zinc were elevated in *E. etunicatum*-treated plants. Overall, persimmon plants inoculated with *E. etunicatum* performed better than those inoculated with *G. clarium*. These results indicate that while both mycorrhizal species are effective, *E. etunicatum* demonstrates superior efficacy in enhancing growth, physiological performance, and tissue nutrient concentration in *D. kaki* under saline conditions.

## 5 Conclusions

This study evaluated the performance of *D. kaki* inoculated with *G. clarium* and *E. etunicatum* under varying NaCl concentrations (0, 50, 75, 100, and 150 mM). *D. kaki* rootstock is an excellent choice due to its good graft compatibility with various cultivars and moderate salt tolerance. This study confirms the suitability of mycorrhizal inoculations for preventing production losses due to increasing soil salinity in Mediterranean regions regarding the results obtained from this study (greenhouse-based, controlled conditions). The performance of *G. clarium* and *E. etunicatum* mycorrhizae under high salt stress was evaluated, and our results show that *E. etunicatum* performed significantly better. Notably, mycorrhizal-inoculated persimmon plants responded much better in terms of phosphorus (P) uptake, with *E. etunicatum* outperforming *G. clarium*. However, since the plants in this study were propagated from seeds—as is common in commercial production—these primarily results demonstrate that *E. etunicatum* can be practically used to enhance salt tolerance in *D. kaki* rootstocks. Nevertheless, the generalizability of these results to practical applications necessitates further research under heterogeneous terrain and soil conditions.

## Data Availability

The raw data supporting the conclusions of this article will be made available by the authors, without undue reservation.
